# Mind the gap: State-of-the-art technologies and applications for EEG-based brain–computer interfaces

**DOI:** 10.1063/5.0047237

**Published:** 2021-07-20

**Authors:** Roberto Portillo-Lara, Bogachan Tahirbegi, Christopher A. R. Chapman, Josef A. Goding, Rylie A. Green

**Affiliations:** Department of Bioengineering, Imperial College London, Royal School of Mines, London SW7 2AZ, United Kingdom

## Abstract

Brain–computer interfaces (BCIs) provide bidirectional communication between the brain and output devices that translate user intent into function. Among the different brain imaging techniques used to operate BCIs, electroencephalography (EEG) constitutes the preferred method of choice, owing to its relative low cost, ease of use, high temporal resolution, and noninvasiveness. In recent years, significant progress in wearable technologies and computational intelligence has greatly enhanced the performance and capabilities of EEG-based BCIs (eBCIs) and propelled their migration out of the laboratory and into real-world environments. This rapid translation constitutes a paradigm shift in human–machine interaction that will deeply transform different industries in the near future, including healthcare and wellbeing, entertainment, security, education, and marketing. In this contribution, the state-of-the-art in wearable biosensing is reviewed, focusing on the development of novel electrode interfaces for long term and noninvasive EEG monitoring. Commercially available EEG platforms are surveyed, and a comparative analysis is presented based on the benefits and limitations they provide for eBCI development. Emerging applications in neuroscientific research and future trends related to the widespread implementation of eBCIs for medical and nonmedical uses are discussed. Finally, a commentary on the ethical, social, and legal concerns associated with this increasingly ubiquitous technology is provided, as well as general recommendations to address key issues related to mainstream consumer adoption.

## I. INTRODUCTION

**FIG. 1.**
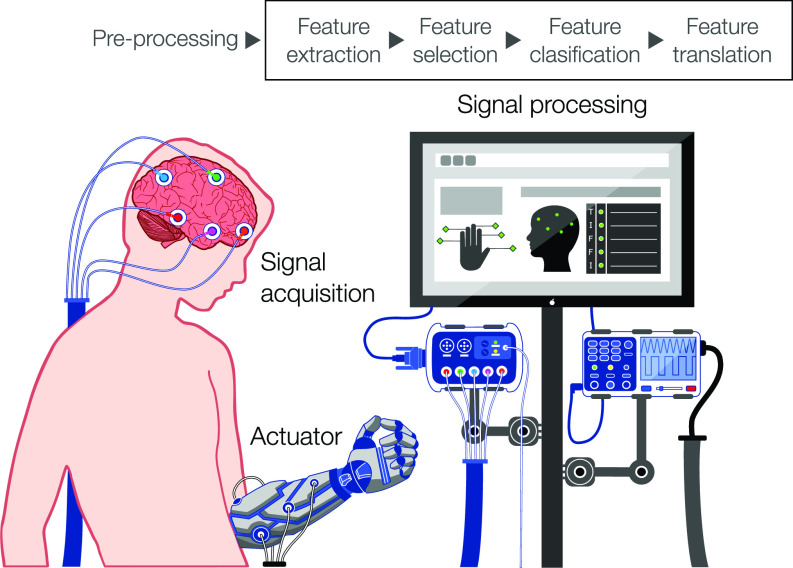
The architecture of an EEG-based brain–computer interface. The schematic depicts the main stages involved in eBCI operation. Noninvasive EEG sensors are used for the acquisition of electrical signals generated by neurons in the cerebral cortex. EEG signals are either acquired from the spontaneous endogenous activity of the brain or evoked by exogenous stimuli. Raw signals are preprocessed, and features are then extracted, selected, classified, and translated to decode user intent. Digital commands are then used to drive different output actuator devices, such as prostheses, exoskeletons, vehicles, or assistive software.

The use of technology to bridge the gap between the human mind and the external environment is no longer restricted to science fiction and is now a reality that is permeating our everyday life. This scientific feat has been made possible through the development of brain–computer interfaces (BCIs), which are systems that integrate hardware and software to establish a direct communication channel between the brain and its surroundings.^1^ BCIs operate by acquiring signals produced by the electrophysiological and hemodynamic activity of the brain in response to different stimuli and specific tasks carried out by the user.^2^ This information is then relayed to effector devices that bypass normal neuromuscular outputs through a series of components that perform sequential tasks, including signal acquisition, signal processing (i.e., preprocessing, feature extraction, and feature classification), signal translation, and device output (Fig. 1). Since their inception in the early 1970s, BCIs have been increasingly used to assess, augment, assist, replace, and restore cognitive and sensorimotor functions, not only in severely disabled patients but also in healthy individuals.^3–8^ Furthermore, the continued advancement of biosensing technologies has driven the rapid translation of BCIs, which have migrated beyond the laboratory toward real-world environments and mainstream applications.^3,9^

Multiple classification systems for BCIs have been proposed based on different criteria, such as the degree of invasiveness, the level of control exerted by the user, and the different types of stimuli used to trigger brain activity.^9,10^ For instance, BCIs can be classified based on the type of brain signals they record into active, reactive, and passive BCIs.^11^ Active BCIs rely on intentional tasks [e.g., finger tapping, mental arithmetic, motor imagery (MI), or music imagery] to trigger brain activity, objectively and without the need for external stimuli. In contrast, reactive BCIs rely on different types of exogenous cues (e.g., audio-visual, interrogative, or painful stimuli), while passive BCIs record arbitrary and unintentional brain signals related to the mental state of the user (e.g., vigilance, drowsiness, or fatigue). Similarly, BCIs have also been divided into two operation modes, depending on whether the presentation of an external stimulus is required or not, termed synchronous and asynchronous, respectively.^12^ In synchronous or “cue-paced” BCIs, the interaction with the system occurs at a fixed time window, and thus, the user is not able to select commands at their own convenience. In contrast, asynchronous or “self-paced” BCIs provide more natural interaction by allowing users to communicate with the system at any time through the continuous processing of brain signals.^9,12^ Although these classification systems have been widely used in the literature, they are hindered by the lack of clear neuroscientific definitions and unified criteria for evaluation, which often prevents the comparison between different studies.^13,14^

A more objective classification system is based on the need for surgically implantable electrodes to monitor brain activity, which categorizes BCIs into invasive, semi-invasive, and noninvasive.^15^ Invasive BCIs are characterized by the use of intraparenchymal grids of microelectrodes, which provide fast information transfer rates (ITRs) and the highest spatial and temporal resolution.^15–17^ Intracortical electrodes allow the recording of three types of brain signals, which are single-unit activity (SUA), multiunit activity (MUA), and local field potentials (LFPs). However, despite their high accuracy and optimal signal fidelity, the risks associated with the surgical procedures largely restrict their use outside well-controlled laboratory and clinical environments. Furthermore, the growth of connective tissue and scar formation around the electrodes following long-term implantation often causes signal deterioration and can lead to device failure.^1,5^ Alternatively, semi-invasive BCIs allow the recording of electrocorticographic oscillations via epidural or subdural electrodes placed on the surface of the cortex.^18^ BCIs based on electrocorticography (ECoG) constitute a less invasive approach that delivers high signal fidelity and amplitude, as well as increased resistance to artifacts caused by blinks or eye movements.^19^ However, despite the lower clinical risk and robust performance over extended recording periods, the need for craniotomies for electrode implantation remains.

**FIG. 2.**
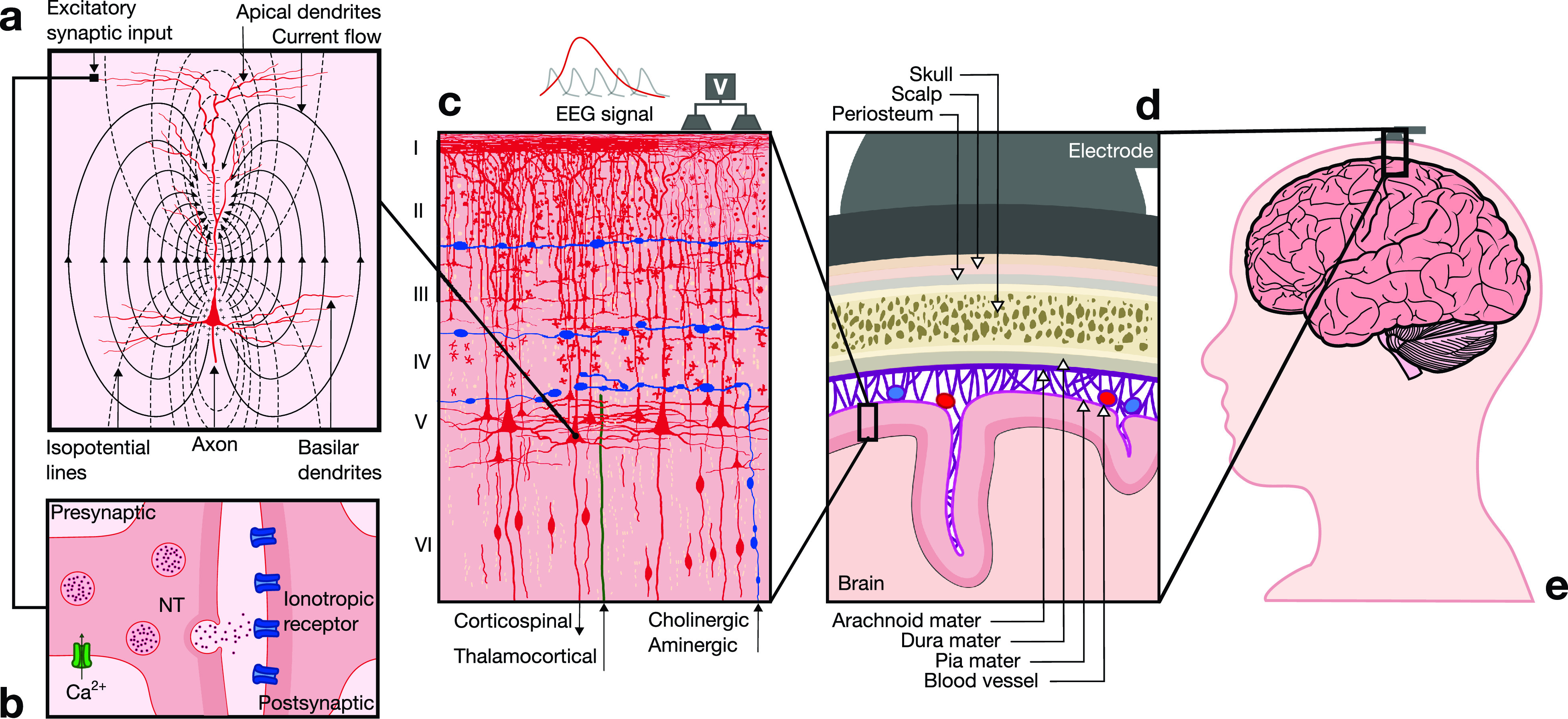
Neurophysiological basis of EEG. (a) EEG signals reflect electrical brain activity that arises from the synchronous activation of groups of pyramidal neurons in the cerebral cortex. Excitatory postsynaptic potentials (EPSPs) generate dipoles by creating separation of charge perpendicular to the surface of the cortex. (b) Communication between neurons is mediated at the synapse. The arrival of an action potential at the presynaptic terminal leads to vesicular release of a neurotransmitter (NT) into the synaptic cleft, which then diffuses to reach membrane receptors on the postsynaptic terminal and trigger an EPSP. (c) The cerebral neocortex is organized in six layers (I–VI) with different cytoarchitectural characteristics. The majority of EEG signals are generated by pyramidal neurons located primarily in layers III and V. These neurons are spatially aligned perpendicular to the cortical surface, which yields a dipole layer orthogonal to the surface of the scalp. EEG activity is measured as differences in voltages recorded at different locations on the scalp, which constitute the summation of postsynaptic potentials from thousands of neurons near each recording electrode. (d) To reach scalp electrodes, EEG signals cross several layers of non-neural tissues with different conduction properties that attenuate the signal. (e) Electrodes are positioned on the scalp in defined configurations, which depend on the functional area of the cortex that is monitored to drive eBCI control.

Although invasive and semi-invasive approaches provide accurate information regarding cortical neurodynamics, the potential benefits of improved signal integrity are outweighed by the surgical risks in the context of everyday applications. Because of this, a variety of noninvasive brain imaging techniques have been developed, which monitor the metabolic [e.g., functional magnetic resonance imaging (fMRI)], magnetic (i.e., magnetoencephalography), and electrical [i.e., electroencephalography (EEG)] activity of the brain.^4,8,9^ In general, noninvasive techniques provide minimal risk and high convenience by enabling the recording of brain signals from the external surface of the scalp. Among these, EEG constitutes the most widely used approach for BCI development, owing to its relatively low cost, high temporal resolution, and high portability and convenience. This technique allows the monitoring of brain activity caused by the flow of ion currents triggered by synaptic activation of neurons in the cortex (Fig. 2).^9,20^ To date, numerous groups have reported the development of EEG-based BCIs (eBCIs) for a wide spectrum of biomedical applications, including the operation of external devices, environmental control, and interface interaction.^21^ Furthermore, recent advancements in EEG signal processing and the development of user friendly and wearable EEG (wEEG) headsets have enabled the migration of eBCIs into mainstream environments for everyday use. In turn, this rapid translation is beginning to reshape human–machine interaction across multiple industries, such as health care and well-being, entertainment, security, education, and marketing.

Herein, state-of-the-art technologies and applications of eBCIs in medical and nonmedical contexts are reviewed. For this, an overview of the neuroanatomical and neurophysiological mechanisms that underlie the generation of electrical brain activity is provided. Recent advancements in wearable biosensing are then discussed with a focus on novel electrode interfaces for long-term and noninvasive EEG monitoring. Commercially available EEG platforms are surveyed to provide a comparative analysis in terms of electrode type and density, functionality, portability, and device performance in the context of eBCI development. Future trends toward the widespread implementation of eBCIs in neuroscientific research are discussed in the context of emerging applications, including automation, entertainment, affective computing, theranostics, and communication. To conclude, a commentary on ethical, social, and legal concerns associated with this increasingly ubiquitous technology is provided, as well as general recommendations to address key issues brought about by mainstream consumer adoption.

## II. NEUROPHYSIOLOGICAL BASIS OF EEG

Neurons are highly specialized cells that constitute the core computational units of the brain. They exhibit a complex polarized structure, which is characterized by a cell body called the soma, as well as two types of cytoplasmatic prolongations, the dendrites that converge on the soma, and the axon that conveys activity away from the soma [Fig. 2(a)]. It has been estimated that the human brain contains approximately 86 × 10^9^ neuronal cells,^22^ with pyramidal neurons comprising about two-thirds of all neurons in the cerebral cortex. Moreover, each pyramidal neuron establishes an average of 7000 synaptic connections.^23^ Synaptic communication can take place at electrical synapses via gap junctions, which interconnect large populations of neurons to synchronize their responses.^24^ Alternatively, transmission can occur at chemical synapses through the vesicular release of neurotransmitters (NTs), which is triggered by the arrival of an action potential at the presynaptic terminal and the subsequent opening of voltage-gated Ca^2+^ channels [Fig. 2(b)]. NTs then diffuse across the synaptic cleft and bind to specific receptors on the postsynaptic terminal, which triggers the opening of ionic channels that modulate membrane potential. Binding of excitatory NTs (e.g., glutamate) triggers excitatory postsynaptic potentials (EPSPs) that cause membrane depolarization, while inhibitory NTs [e.g., gamma-amino butyric acid (GABA)] trigger inhibitory postsynaptic potentials (IPSPs) and membrane hyperpolarization.^23,24^

The neocortex is a 2–5 mm thick layer of cells on the outermost surface of the brain, which is comprised of functionally distinct motor areas that control voluntary movement, sensory areas that receive and process information from the senses, and association areas that mediate higher-order cognitive functions. Based on differences in cytoarchitectural characteristics, the cerebral neocortex is organized in six layers (I–VI) comprised of different cell types that vary across the cortex and have been defined in spatial arrangements for specific functions [Fig. 2(c)]. For instance, pyramidal neurons constitute the main cell type in layers III and V, exhibiting particularly large morphologies in layer V of the motor cortex, where they project apical dendrites all the way to layer I. Excitatory synapses are usually located on dendritic spines along superficial laminae, while inhibitory synapses are mainly found on the soma in deeper laminae.^24^ Because of this highly organized columnar organization, the arrival of EPSPs or IPSPs generates a superficial area of extracellular negativity, known as a sink, and a basal area of extracellular positivity, known as the source. This separation of charge is referred to as a dipole, which is orthogonally oriented to the surface of the scalp [Fig. 2(a)]. However, dipoles from individual neurons are too small and the electrical signals must travel across several layers of non-neural tissues with different conduction properties [Fig. 2(d)]. As a result, the simultaneous activation of thousands of neurons is necessary to generate a signal that is strong enough to be detected by electrodes on the scalp.^25^

EEG signals are field potentials that arise from the synchronized synaptic activity of pyramidal neurons, which are organized in columns perpendicular to the cortical surface. In particular, the synchronous activation of approximately 100 cortical neurons in an area of at least 6 cm^2^ is required to produce a signal that could be recorded on the scalp.^26^ The summation of these individual dipoles is measured by scalp electrodes as a single dipole with a magnitude that reflects the number of neurons involved. Graphically, the EEG is a dynamic representation of the differences in voltage between an active electrode above the site of neuronal activity and a reference electrode.^24^ Surface EEG measures the activity of cortical neurons located just below the scalp, and thus, the electrical contribution of deeper structures such as the thalamus, hippocampus, or the brainstem is minimal.^27^ However, input from distal sites exerts a significant influence on the modulation of EEG signals. For instance, the dorsal thalamus is considered as the main subcortical generator of EEG rhythms, which acts by synchronizing the activity of neocortical neurons. Once they are generated, EEG signals must travel from the cortex, through the meninges, the skull, and the scalp to reach the electrodes [Fig. 2(e)]. The voltage measured at the scalp cycles between positive and negative values at a rate that constitutes the frequency of the signal. The typical frequency bands recorded by EEG range from 0.1 Hz to around 30 Hz, since higher frequencies are heavily attenuated by the skull and the scalp or obscured by artifacts. Finally, after the signals have been acquired by the electrodes, these are processed and translated into meaningful commands to enable eBCI control.

## III. FUNDAMENTALS OF eBCI DESIGN AND OPERATION

The operation of eBCIs relies on patterns of brain activity that are triggered by different stimuli or tasks, thus enabling the control of external devices. To achieve this, specific features contained in analogue brain signals need to be extracted, classified, and translated into digital commands in a series of sequential stages (Fig. 1). In the signal acquisition stage, brain signals are recorded using electrodes positioned on the scalp in defined configurations, such as the international 10–20 system standardized by the American Electroencephalographic Society.^28^ EEG signals are highly susceptible to multiple sources of noise, and thus, raw data are first transformed in a preprocessing stage to enhance signal quality and improve the accuracy of the system. This is mainly done through linear and nonlinear filtering techniques that eliminate noise and known physiological or external artifacts.^29–31^ In the feature extraction stage, discriminative and nonredundant information is identified and extracted from EEG signals to yield a set of features on which classification can be performed. Multiple feature extraction techniques have been reported in the literature, which are based on different approaches, such as dimension reduction,^32–36^ space domain,^37–39^ and time-frequency domain.^40–43^ Although a large number of features can be retrieved from multiple channels and time segments, extracted features should capture salient signal characteristics in order to differentiate between task-specific brain states.^44^ Therefore, some systems incorporate a stage of feature selection in which only the most discriminant features in a given set are communicated to the classifier, which reduces computational complexity and processing times.^21^ In the classification stage, the type of mental task carried out by the user is decoded based on the selected features using different classification algorithms, including Bayesian analysis,^45,46^ k-nearest neighbor classifiers,^47,48^ linear discriminant analysis,^49,50^ support vector machine,^51,52^ and artificial neural networks.^53,54^ Finally, in the signal translation stage, the classified features are passed to a translation algorithm that converts them into instructions. Typical instructions can range from letter selection and cursor control to commands for the operation of robotic vehicles and prostheses. In this regard, the complexity of the actuator and the capacity of the user to produce the control signals required for device operation play a significant role in the design of eBCIs.

Although the high temporal resolution of EEG signals is remarkably advantageous for BCI operation, they also present several challenges related to their nonstationarity and high susceptibility to noise and signal artifacts.^21^ This is particularly relevant for applications based on online (real-time) operation where EEG signals need to be accurately processed within limited time windows. Because of this, real-time signal processing techniques have been often associated with trade-offs between speed and accuracy, while the latency, consistency, and flexibility of these approaches remain a concern.^55,56^ In recent years, artificial intelligence (AI) and machine learning have been increasingly used to automate, complement, and enhance the analysis of big data for biomedical applications, including the processing and interpretation of EEG signals.^57,58^ In particular, approaches based on deep learning have shown great promise to streamline the EEG signal processing pipeline, since they can be used to perform feature extraction, selection, and classification within a single processing block.^59,60^ Therefore, apart from more capable and versatile biosensing hardware, the need for robust and efficient tools for signal processing constitutes one of the main challenges for the success of eBCI technology. For a more in-depth discussion on state-of-the-art EEG signal processing techniques, readers are directed to comprehensive reviews on this subject.^58,61–63^

### A. General considerations for eBCI design

The remarkably wide range of eBCI effector outputs has brought forth the development of multiple control strategies, which vary according to the capabilities and limitations of the end-user, as well as the intended application of the device. Furthermore, these considerations need to be taken into account from the early stages of the design process to ensure optimal device compatibility and functionality. For instance, the multidimensionality of the system, which refers to the need for multiple elements of control (i.e., degrees of freedom), could constitute a significant limitation for user adoption. Previous works have shown that the control of a computer mouse typically requires 2–4 degrees of freedom, while achieving full dexterity over the individual joints of a robotic hand could require as many as 22.^5^ To circumvent this limitation, bidirectional BCI systems have been developed, which allow sensory information to be directly input to the nervous system via different feedback mechanisms, including brain stimulation,^64^ reaction force,^65^ and somatosensory stimulation.^66^ These closed-loop BCIs constitute co-adaptive systems where the user and the computer learn from each other, which facilitates the operation and assimilation of complex neuroprosthetics.^67,68^

The design process of eBCIs should take into account the physical capabilities and the requirements of the end-user. For instance, users may require continuous control over the system, and thus, interfaces based on discrete selection methods and synchronous operation may not provide adequate response times. In addition, a robust strategy for the detection of noncontrol states should be implemented, which allows users to focus on activities unrelated to eBCI operation. For this, systems could be designed to differentiate control and rest signals in order to tolerate periods of inactivity, or they could incorporate brain-controlled switches that enable or disable device control at will.^13,69,70^

Another aspect to consider is the level of control, which refers to the way in which device outputs are determined by user inputs. For instance, the nonstationary nature of EEG signals often hinders the ability to determine user intent consistently over extended periods of time.^71^ Therefore, systems often need to be capable of carrying out extensive signal smoothing and intelligent processing to translate inputs into safe and effective actuator outputs. Alternatively, systems have been designed so users are in charge only of higher-level abstract operations while semi-autonomous subsystems manage other aspects of low-level device control.^64^ More recently, intelligent entities for shared control have been developed, which assist users in accomplishing a desired task with varying degrees of automation.^72–74^ Furthermore, the cumbersome training that is required to operate multidimensional eBCI systems could prevent their use by a significant population of users, a phenomenon that has been referred to as BCI illiteracy.^75^ To circumvent this limitation, hybrid systems that combine inputs from multiple types of sensors have been developed.^76,77^ These hybrid systems have been shown to provide higher ITRs, lower false positive rates, and enhanced man-machine adaptability, when compared to conventional single-mode BCIs.^78^ Finally, apart from considerations related to hardware and software components, one critical aspect of eBCI design corresponds to the selection of a system paradigm. Paradigms refer to the overall approach that is used to elicit the brain signals that drive eBCI operation, and thus, they determine the control strategy of the device.

### B. Overview of eBCI paradigms

The high temporal resolution of EEG allows the identification of a variety of brain signals that are representative of different aspects of user intention, attention, and perception. In turn, these patterns of brain activity can be modulated through their association with a particular sensory, cognitive, or motor process carried out by the user. In the context of eBCIs, this targeted modulation of brain signals for device control is carried out through various system paradigms.^64,68^ Similar to the design of the physical hardware, the choice of system paradigm requires careful consideration of the psychological and physical conditions of the user, environmental limitations, and the level of accuracy required.^79^ To date, several paradigms have been reported in the literature, which vary according to the type of brain signals used for device control, namely, evoked or spontaneous signals.^9^

Evoked potentials (EPs) are involuntary time-locked deflections on brain activity that occur in response to the presentation of an external event.^4^ Among these, visual and P300 EPs constitute two of the most widely used control signals in eBCI paradigms.^80^ For instance, steady-state visual evoked potentials (SSVEPs) are triggered by an oscillating visual stimulus (e.g., a flashing light) presented at a fixed frequency and are recorded with maximum amplitude over the occipital region.^20,81^ Although they enable relatively accurate and rapid command input, SSVEPs rely on normal attentional capacity and oculomotor function, which are often compromised in patients with severe neurological disease. On the other hand, P300 EPs are positive deflections that occur approximately 300 ms after the presentation of an oddball stimulus (i.e., an unexpected and random variation within a regular pattern of stimuli).^1,80^ P300 signals could be evoked by the presentation of visual, auditory, or somatosensory stimuli and they can be recorded over the central and parietal regions.^20,80^ The generation of P300 EPs requires little training by the user and the amplitude of the response peak could be increased as the stimulus becomes less probable.^9^ However, different sampling, averaging, filtering, and eye-artifact removal techniques are often needed to isolate the signal, since EP amplitudes are significantly smaller than other signals acquired during EEG recording.^4^

Paradigms based on spontaneous signals do not require external stimuli to produce control actions, since they rely on changes in EEG activity elicited by mental tasks. EEG activity can be classified depending on the predominant frequency (ƒ) of the signal into delta (ƒ < 4 Hz), theta (4 Hz < ƒ < 7 Hz), alpha (8 Hz < ƒ < 12 Hz), beta (12 < ƒ < 30 Hz), and gamma (ƒ >30 Hz) bands.^21^ In particular, alpha band activity from the sensorimotor cortex is designated mu or Rolandic rhythm, and oscillations occurring in this and the beta bands are collectively called sensorimotor rhythms.^21,82^ The planning, execution, and completion of voluntary movements elicit power decreases or increases in sensorimotor rhythms that are referred to as event-related desynchronization (ERD) and synchronization (ERS), respectively.^1,80^ Paradigms based on ERD and ERS do not require the presentation of external stimuli, since brain signals are modulated through mental tasks, such as MI. MI refers to a dynamic mental state during which the representation of a movement is imagined without overt motor output.^83,84^ As such, MI paradigms are widely used for the control of assistive eBCIs for paralyzed individuals that retain the capacity to conceive limb motion.^85^ However, the operation of these systems requires extensive user training and they often exhibit high variability in performance and poor accuracy.^4,86^ Another source of spontaneous signals is slow cortical potentials (SCPs), which are shifts in cortical activity that range from several hundred milliseconds to several seconds and occur at a frequency below 1 Hz.^87^ SCPs can be self-regulated by healthy and paralyzed users through operant training, and paradigms based on these signals are widely used for the control of assistive communication devices.^1,88^ Furthermore, nonmotor cognitive tasks such as visual counting, mathematical computations, mental rotation, or music imagery could be used to generate SCPs by users with severe motor impairment.^9,89^

In general, evoked signals allow higher throughputs with comparatively easier training and operation than spontaneous signals, which require sustained user attention and concentration. Similar to hybrid systems that integrate different brain imaging techniques, the combination of multiple control signals has also been explored to circumvent the limitations of individual single-mode systems.^77,78,90–92^ Hybrid approaches enable a higher number of control commands, as well as improved performance in terms of classification accuracy and signal detection times. However, they also require significantly more complex system architectures, which could hinder user acceptability. Therefore, as systems become increasingly more elaborated, hardware complexity remains a significant hurdle for the development of portable, wearable, and low-cost platforms. With regard to eBCIs, EEG sensors constitute the most crucial component since they determine the resolution of brain pattern recognition, while also influencing ease of use and user adoption.

## IV. STATE-OF-THE-ART ELECTRODE TECHNOLOGIES FOR eBCIs

**FIG. 3.**
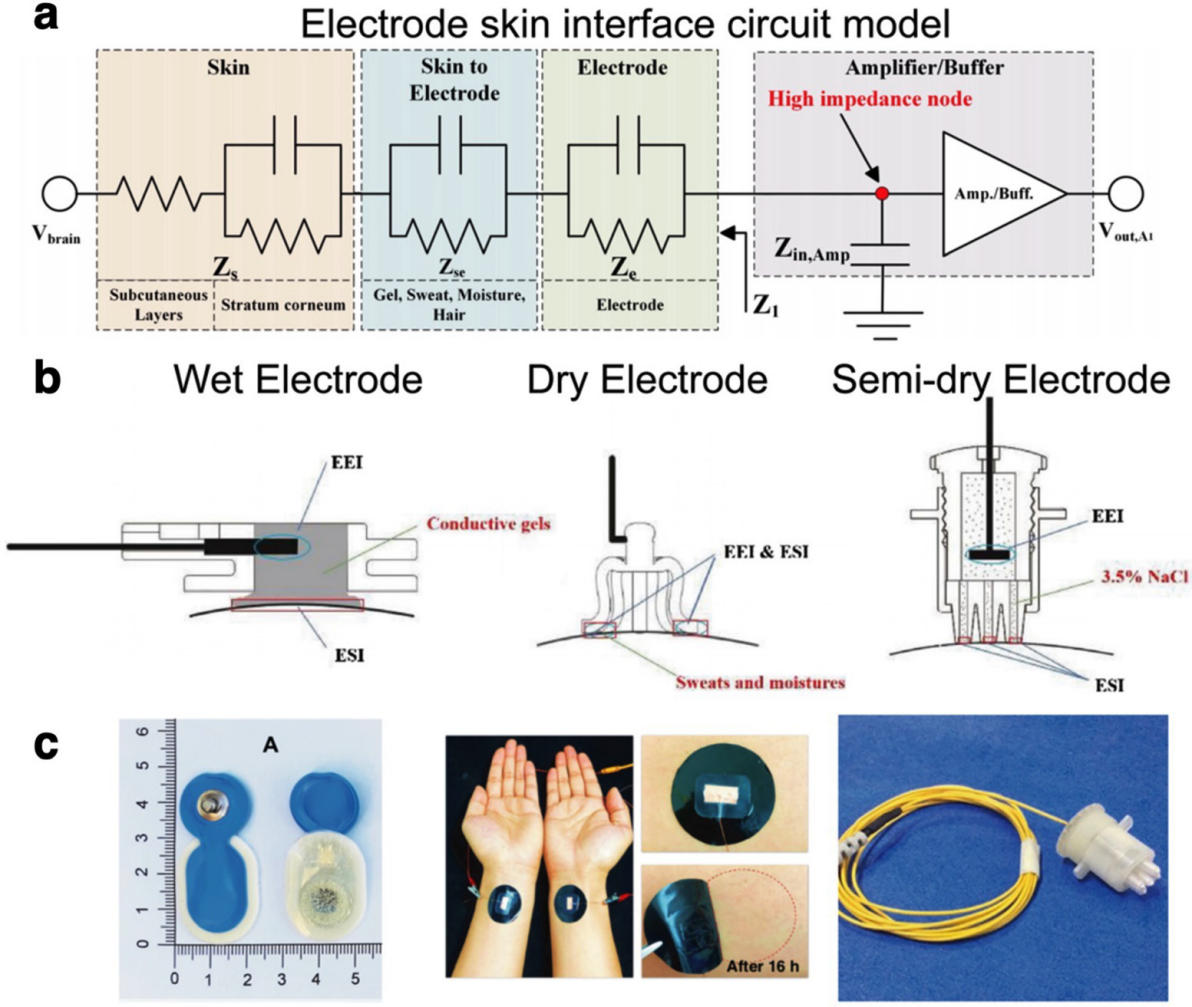
(a) Equivalent circuit model of the interface formed between an electrode and the skin. Reproduced with permission from Shad *et al.*, IEEE Sens. J. **20**(24), 14565–14577 (2020). Copyright 2020 Authors, licensed under a Creative Commons Attribution (CC BY) license.^93^ (b) Schematic of the differences in electrode contact on the scalp among wet (left), dry (middle), and semi-dry (right) electrodes, highlighting the electrode–skin interface (ESI) and the electrode electrolyte interface (EEI). Reprinted with permission from Li *et al.*, Sens. Actuators, B **277**, 250–260 (2018). Copyright 2018 Elsevier.^95^ (c) Representative images of the different types of skin interface electrodes (wet—left, dry—middle, and semi-dry—right). Left panel reproduced with permission from Leach *et al.*, Front. Neurosci. **14**, 586 (2020). Copyright 2020 Authors, licensed under a Creative Commons Attribution (CC BY) license.^98^ Middle panel reproduced with permission from Zhang *et al.*, Nat. Commun. **11**(1), 4683 (2020). Copyright 2020 Authors, licensed under a Creative Commons Attribution (CC BY) license.^115^ Right panel reprinted with permission from Li *et al.*, Sens. Actuators, B **237**, 167–178 (2016). Copyright 2016 Elsevier.^125^

Due to the relative low costs and ease of use of surface EEG, this technology holds great potential to be commercialized for the general public. Despite multiple advancements in computational intelligence and signal processing techniques, the engineering of accurate and user-friendly sensors remains a critical issue for the development of consumer-grade wEEG platforms. Successful recording of the small current flows that comprise an EEG relies heavily on the front-end hardware establishing a low impedance interface with the scalp. This is mainly because high interfacial impedances decrease the quality of the recorded signal while also increasing signal noise. Although the presence of physiological high-impedance layers such as the skull greatly attenuates signal amplitude, primary loss of signal comes from the interfacial impedance established between the electrode surface and the scalp. An equivalent-circuit model [Fig. 3(a)] can be used to visualize the signal flow between the physiological environment and the conductive electrode material.^93^

In an ideal situation, the interfacial impedance would be reduced to a minimal value to enable direct coupling of the recording electrode to the scalp. As such, minimizing this interfacial impedance is the primary design criteria for the development of EEG electrodes. In a clinical setting, this typically involves preparation of the skin by degreasing and abrasion to remove the stratum corneum (SC) at the chosen electrode sites. The SC accounts for 200 KΩ cm^2^ to 200 Ω over a respective frequency range of 1 Hz–1 MHz, making it one of the major contributors to the overall skin impedance.^94^ Traditionally, a conductive paste or gel is then applied to the electrode site to bridge the skin–electrode interface and lower interfacial impedance. In this regard, noninvasive electrode interfaces have customarily been classified depending on the presence of electrolytes at the electrode–skin interface (ESI) into wet, dry, and semi-dry electrodes.^95^ EEG electrodes can be categorized as either active or passive. Active electrodes have preamplification circuits built into the electrode, thus minimizing the impact of noise from ambient electrical activity during transmission along the electrode lead. Active preamplification can be applied to wet, dry, and semi-dry electrodes; however, it will add to the weight, size, and cost of the electrodes compared to their passive counterparts.^96^

### A. Wet, dry, and semi-dry electrodes

Wet electrodes form a “wet” interface with the scalp through the use of a conductive gel or paste (Fig. 3). The addition of this conductive element to the electrode–scalp interface creates a low impedance pathway that effectively reduces the contact impedance.^93,97,98^ Moreover, a robust connection to the scalp is formed, owing to the surface conformation properties of the wet conductive element.^95^ Wet electrodes are commercially available in a range of designs and materials (Table I). Tallgren *et al.* conducted an evaluation of commercially available electrodes (silver, tin, and gold cup electrodes; sintered Ag/AgCl, platinum, and stainless-steel electrodes; and disposable Ag/AgCl) and gels (nine conductive gels/pastes) for recording of slow EEG potentials.^99^ Their results showed that the choice of both components was critical for direct current recording of EEG potentials. Notably, reusable sintered Ag/AgCl electrodes demonstrated excellent DC-stability, low resistance, and minimal low-frequency noise. Tin and stainless-steel electrodes were found to have the highest noise levels.

**TABLE I.** Comparative summary of electrode designs.

Electrode	Material	Advantages	Disadvantages
Wet			
Pad	Metal^a^, Ag/AgCl coating, sintered Ag/AgCl	Low impedance	Long setup requiring technician
			Drying
			Shorting
			Irritation
Cup	Metal	Low impedance	Long setup requiring technician
		Less prone to lift off	Shorting
		Large cavity for electrolyte reduces effects of drying	Irritation
Pregelled	Metal or Ag/AgCl with electrolyte	Low impedance	Single use
		Faster setup	Drying
			Shorting
			Irritation
Solid-gel	Hydrogel with electrolyte	Low impedance	Drying still an issue
		Faster setup	
		Less irritation	
		Less prone to shorting	
		Slower drying	
Semi-dry			
Gel reservoir	Metal or Ag/AgCl with electrolyte	Faster setup	Higher impedance than wet electrodes
		Less prone to shorting	Drying still an issue
Dry			
Thin film	Metal, Ag/AgCl coatings	Fast setup	Increased impedance.
			Poor conformability
			Discomfort
Spikes, bristles, needles	Metal, conductive composites	Fast setup	Discomfort
		Geometries help push past hair	Increased impedance
		Improved skin contact	
		Reduced motion artifacts	
Capacitive (noncontact)	Metal or Ag/AgCl with dielectric/air gap	Not as dependent on skin contact	
Dry foam	Conductive composites	Comfort	Higher impedance
Conductive fabric	Metal coatings, organic conductor coatings	Improved conformability and comfort	Poor skin contact. Prone to motion artifact
		Ideal for active use	

^a^ Metal = Au, Ag, Sn, and stainless steel.

The ability to readily form a low impedance connection has made wet electrode technology the clinical gold-standard.^100^ However, wet electrodes are also prone to the degradation of the electrolyte interface over time, which restricts their use to applications where only short-term monitoring is needed.^101^ The application of multichannel wet electrodes generally requires a trained technician and restricts movement of the subject, thus limiting their application to clinical settings. Furthermore, the use of abrasive and conductive gels can cause skin irritation and discomfort.^102,103^ Wet electrodes also suffer from special limitations, due to the risk of conductive gel bridging two electrode sites and causing a short-circuit. These limitations significantly reduce the applicability of wet electrode interfaces for long-term or nonclinical EEG recordings, as needed by many eBCIs. To this end, current research on wet electrodes is focused on the stabilization of the electrolyte interface. Solid–gel electrodes address issues related to the placement and drying of wet electrodes by improving moisture retention through the incorporation of a hydrophilic hydrogel network.^104,105^ The hydrogel, which replaces the typical buffer between the skin and electrode, is swollen in an electrolyte. The soft hydrogel can also deform to the skin, maintaining good physical contact and reducing interfacial impedance. One of the most promising approaches for creating long-term stable wet electrodes is the incorporation of ionic liquids (ILs) into a long-lasting gel interface.^106,107^ Owing to their unique chemical properties, ILs do not suffer from the limitations of other types of liquids, such as evaporation. Although the long-term toxicity of many ILs remains a concern, new IL-based gels have been developed with significantly improved biocompatibility.^107,108^

Dry electrodes form an interface with the scalp without the use of any conductive electrolyte solution (Fig. 3). The majority of modern wEEG systems have focused primarily on the use of dry electrodes due to their relative ease of application and lack of scalp preparation. Since these electrodes do not benefit from the excellent interfacial impedance of the electrolyte, the material properties of the electrode become critical to ensure uniform contact with the scalp. Instead of bulky solid metal electrodes, dry interfaces typically employ thin conformal materials to maximize the surface area of the electrode that can access the scalp.^109^ Dry electrodes may be fabricated from thin metal films laminated to a polymeric substrate,^110^ metallic or conductive coatings of nonconductive components, or from conductive composites. Conductive composites consist of conductive particles such as metal^111^ or organic conductors (graphite,^112,113^ carbon nanotubes,^114^ or conductive polymers^115,116^) in an elastomeric matrix.

Alternative approaches to dry electrodes such as temporary tattoo electrodes have been explored. Bareket *et al.* reported the fabrication of transferable, conformal tattoo arrays for surface electromyography (EMG) using standard screen-printing processes coupled with plasma polymerization of poly(3,4-ethylenedioxythiophene) PEDOT.^117^ The tattoo electrodes were found to have similar normalized impedance compared to commercial pregelled Ag/AgCl electrodes. Ferrari *et al.* demonstrated a similar approach using inkjet printing of PEDOT: polystyrene sulfonate dispersions onto a transfer paper substrate.^118^ Electrodes were connected with sputtered-gold tracks and then insulated with an adhesive glue layer. Notably these electrodes were demonstrated to allow growth of hair through the active electrode sites over a 24-hour period with no noticeable effect on the quality of EMG recordings. This approach has considerable potential in the application of long-term monitoring as it may remove the need to continually rehydrate and reposition electrodes. However, typically the surface of the scalp is covered with dense hair and EEG signals are intrinsically weak, which negatively impact the recording quality. Thin film tattoos are not easily applied to the scalp without removal of the hair. To circumvent this limitation, multiple design strategies have been explored with an aim of improving the long-term stability of the electrode–scalp interface, while also enhancing user comfort and compliance for everyday use.

Finger-based sensors with copper pins,^119^ gold-coated spring-loaded pins,^120^ or flexible polymer pins^121^ provide high geometric conformity to the irregular scalp surface, which minimizes electrode impedance. However, despite the incorporation of flexible substrates, previous works have reported that spring-loaded contact pins could lead to user discomfort and even constitute injury hazards in ambulatory or highly dynamic environments.^122^ Furthermore, the intricate structure of these sensors greatly increases the cost and the complexity associated with the manufacturing process. Alternatively, other groups have developed structures that more uniformly distribute the pressure on the skin, in order to minimize the discomfort associated with conventional finger-based sensors. For instance, low-cost electrode brushes comprised of silver-coated polymeric bristles have been reported.^123^ This strategy was shown to provide high redundancy toward maintaining mechanical and electrical contact to the scalp, while also delivering high-quality EEG recordings. In addition to flexible bristles, arched structures that match the curvature of the scalp have also been investigated. Sterling silver electrodes have been recently developed based on a 3D-printed reverse-curve-arch design, which distributes the pressure exerted on the skin and effectively passes through the hair to reach the scalp.^124^ In recent years, several types of dry electrodes with varying geometries and designs have been commercialized, which are generally coupled to EEG amplifiers with very large input impedances or active amplifiers located in close proximity to the electrode itself.

As a compromise between wet and dry interfaces, there has been a recent push toward the use of semi-dry electrodes where a minimal amount of electrolyte is used to improve the interfacial impedance (Fig. 3). This small volume of electrolyte creates an initial seal between the scalp and the dry electrode, minimizing the area exposed to evaporation or degradation.^97,125^ Although the electrolyte does not decrease contact impedance as much as wet electrodes, the extra contact has been shown to significantly improve signal-to-noise ratio (SNR). In addition, semi-dry electrodes provide improved user comfort compared to wet electrodes, where the thick layer of electrolyte needed could lead to skin reactivity over time.^97^ However, despite the small volumes of electrolyte used, issues related to signal degradation still hinder long-term wearability. In this regard, new design strategies for semi-dry electrodes focus on identifying methods to enable the controlled release of electrolyte into the interface for long-term recordings.^126^

Maintaining the long-term stability of the electrode–scalp interface is paramount to the successful implementation of eBCIs. Although all three interface variants have seen significant improvements in their ability to record high-SNR EEG, wet electrodes remain the clinical gold standard. Therefore, there is a need for studies that focus on systematic comparisons between electrode technologies, which could elucidate the technical and scientific advantages that each platform provides. In addition, further multidisciplinary improvements aimed at enhancing the stability, comfort, and performance of dry electrodes are needed. Alternatively, materials-based approaches for epidermal electronics^110,127^ and noncontact capacitive electrodes^128,129^ have been explored to develop EEG systems with enhanced wearability. However, despite the advantages provided by these technologies, the majority of consumer-grade wEEG platforms that have reached the market rely on the use of dry and semi-dry electrodes.

### B. Survey of commercial wEEG platforms

In the context of eBCIs, the selection of wEEG hardware depends on the required type, number, placement, and sampling frequency of the electrodes, as well as the resolution of the signal.^130^ These criteria vary widely depending on the specific application, and thus, multiple wEEG platforms with varying degrees of sophistication are available at different price points. In general, modern wEEG devices have largely focused on limiting the placement of recording units to the head, eliminating the need for bulky instrumentation boxes and restricting the use of wires to minimize noise.^100^ In recent years, the advancement of wireless technology and the emergence of miniaturized and power efficient electronics have enabled the commercialization of a variety of consumer-grade wEEG headsets (Table II).

**TABLE II.** Technical specifications of commercially available wEEG devices.

Device	Electrodes	Channel count	Sampling rate (Hz)	Weight (g)	Battery life (h)	Resolution (bits)	Price (USD)
NeuroSky MindWave Mobile 2	Dry	1	512	90	8	16	$100
Muse 2	Dry	4	220/500	61	5	10	$250
MyndPlay MyndBand	Dry	3	512	300	10	⋯	$270
Emotiv Insight	Semi-dry	5	128	⋯	8	14	$300
Emotiv Epoc X	Saline	14	128/256	170	12	14/16	$850
OpenBCI EEG Electrode Cap	Wet	16	125	⋯	24	24	$1400
Emotiv Flex	Saline/wet	32	128	⋯	9	14	$1700
mBrainTrain Smarting	Wet	24	220/500	60	5	24	$6730
NeuroScan Siesta	Wet	32	1024	300	⋯	16	∼$18 000
Brain Products LiveAmp 64	Dry/wet	64	1000	120	4	24	>$18 000
ABM B-Alert X24	Wet	20	256	110	8	24	∼$20 000
Cognionics Quick 30	Dry	30	500	610	8	24	$22 000
Wearable Sensing DSI 24	Dry	21	300/600	600	8	16	$22 500
g.tec g.Nautilus Research	Dry/wet	64	250	140	6	24	≤$25 000
Neuroelectrics Enobio 32	Dry/wet	32	500	97	6	24	∼$25 000
IMEC EEG headset	Dry	8	256	200	8	12	$25 000
ANT Neuro eggo Sports	Dry/wet	64	2048	500	5	24	∼$50 000
Cognionics Mobile 128	Wet	128	500	460	6	24	≤$50 000

Despite the wide variety of wEEG systems available, further research efforts are needed to produce more capable and user-friendly sensors, as well cost-effective electronics with reduced power consumption. Apart from more capable hardware, highly compelling and engaging applications are critical to promote the use of these technologies outside laboratory environments. With the advent of increasingly sophisticated and affordable devices for wearable biosensing, the development of so-called killer apps will ensure the rapid and widespread dissemination of eBCIs among the general population.

## V. CURRENT and EMERGING APPLICATIONS OF eBCIs

### A. Health care and well-being

**FIG. 4.**
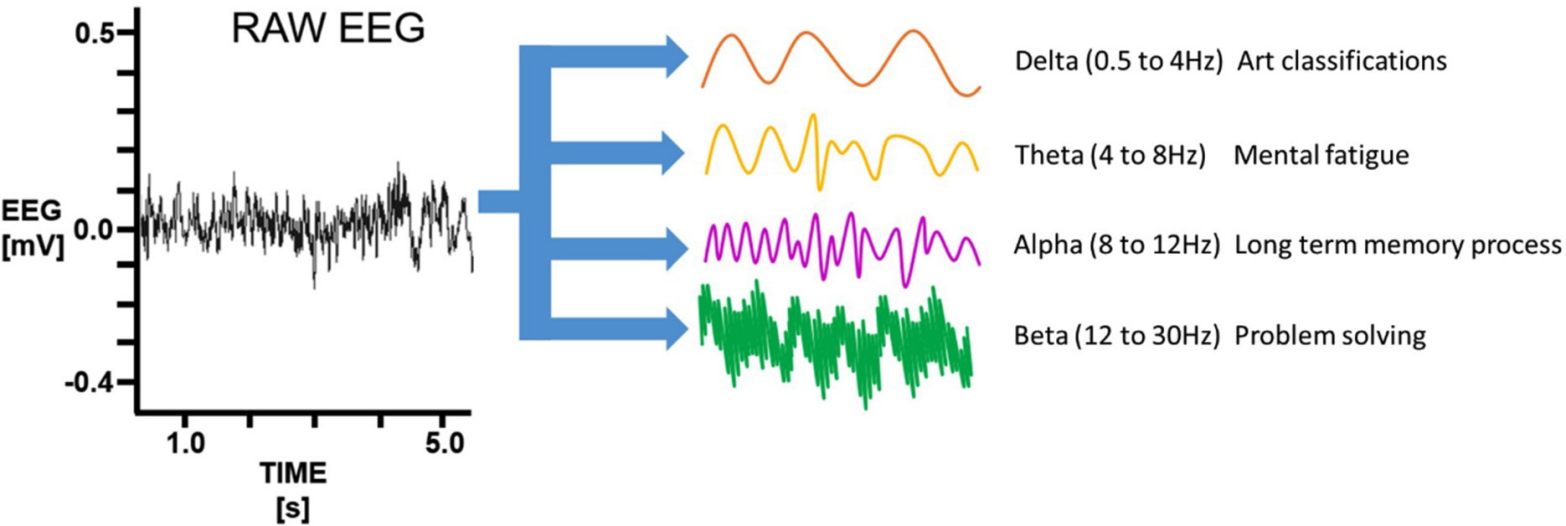
Different frequency bands representative of brain activity. Variations in brain activity detected by EEG are indicative of different aspects of brain function. The schematic shows the raw EEG trace decomposed and reconstituted into delta, theta, alpha, and beta time series traces. Reproduced with permission from Ma *et al.*, PLoS One **15**(6), e0232381 (2020). Copyright 2020 Authors, licensed under a Creative Commons Attribution (CC BY) license.^142^

Traumatic brain injuries (TBI), post-traumatic stress disorder (PTSD), stroke, and other neurodegenerative diseases could impair the function of motor nerves and other structures of the central nervous system (CNS). Therefore, targeting the mechanisms underlying this process could enable the development of rehabilitative and restorative therapies. As described in previous sections, BCIs constitute promising options to develop assistive technologies for patients with impaired motor function.^131–133^ Although brain monitoring techniques such as computed tomography (CT) and fMRI are routinely used to evaluate brain function, their limited temporal resolution does not allow the detection of small disturbances in brain activity.^134^ In this regard, EEG and MI have been widely explored for the rehabilitation of patients suffering from TBIs, stroke, and other neurodegenerative diseases.^135–138^ This is mainly because the high temporal resolution of EEG allows the accurate detection of transient power changes in different frequency bands (Fig. 4), which are triggered by these pathological conditions.^139–141^

One of the most widely reported therapeutic applications of eBCIs is neurofeedback (NFB), which relies on the acquisition and *in situ* processing of EEG signals followed by direct feedback to the patient in real time. This technique allows the self-regulation of different neurophysiological parameters by the user, which translates into self-modulation of brain function and ultimately into behavioral changes.^142^ NFB is widely used for patients that undergo epileptic seizures with high amplitude focal sharp wave discharges, which could be trained to limit rhythms that lead to the generation and propagation of seizures.^143^ The high efficacy of NFB and the emergence of complementary technologies, such as electrooculography (EOG) and electromyography (EMG), have led to the development of multiple applications for this technology.^144^ In addition, other eBCIs based on speech imagery have been used for word recognition and assistive communication devices.^144^ Finally, eBCIs have also enabled the development of prostheses with enhanced functionality, when compared to conventional EMG-based approaches.^145^ Although multi-degrees of freedom (DOF) control remains challenging with conventional techniques, the use of neural network models holds great promise to develop eBCI technologies that improve the operation of complex prostheses.^145^

In addition to therapeutic applications, eBCIs have also been used for diagnostic purposes due to the correlation of alterations in EEG activity with the onset of neurodegenerative disorders. For instance, it has been suggested that a decrease in gamma activity may be directly associated with cognitive decline.^146,147^ Furthermore, previous works have shown that Alzheimer's disease (AD) leads to increases in delta and theta activities, as well as decreases in alpha and beta band activities. It has been demonstrated that EEG-based classification of patients with subjective cognitive impairment (SCI) and AD can be carried out with high accuracy.^148^ This evidence suggests that eBCIs constitute remarkably advantageous diagnostic tools for potential early diagnosis of a variety of neurological disorders.

The continuous monitoring of brain activity provided by EEG holds great promise for the development of technologies aimed at modulating user mood and emotions through drugs, meditation, and other strategies. For instance, pharmaco-EEG refers to the qualitative analysis of the effects of chemical substances on the CNS. Different compounds have been shown to exert varying effects on brain function, including intermixing theta and/or delta activity, increasing beta activity, and decreasing the amplitude and/or frequency of the alpha rhythm.^148^ Therefore, eBCIs could potentially be used to monitor the effect of different drugs on brain function in order to design custom dosage schemes with increased therapeutic efficacy. Alternatively, different types of meditation practices, including focused attention (FA) and open monitoring (OM), could also be used to modulate brain activity. FA and OM have been shown to enhance theta activity in anterior brain regions and alpha activity in posterior brain regions, as well as gamma activity in parietal and occipital regions. Therefore, eBCIs constitute highly versatile alternatives that could enable the therapeutic modulation of brain activity without the need for conventional pharmacological strategies. Although eBCIs have been explored for mood classification based on the detection of changes in brain activity, the remarkable complexity of human emotions still constitutes a significant challenge. However, recent advances in machine learning methods to analyze big data in a comprehensive manner hold great potential for the detection of different emotions such as sadness, disgust, and amusement, among others.

### B. Environmental control, occupational safety, and security

**FIG. 5.**
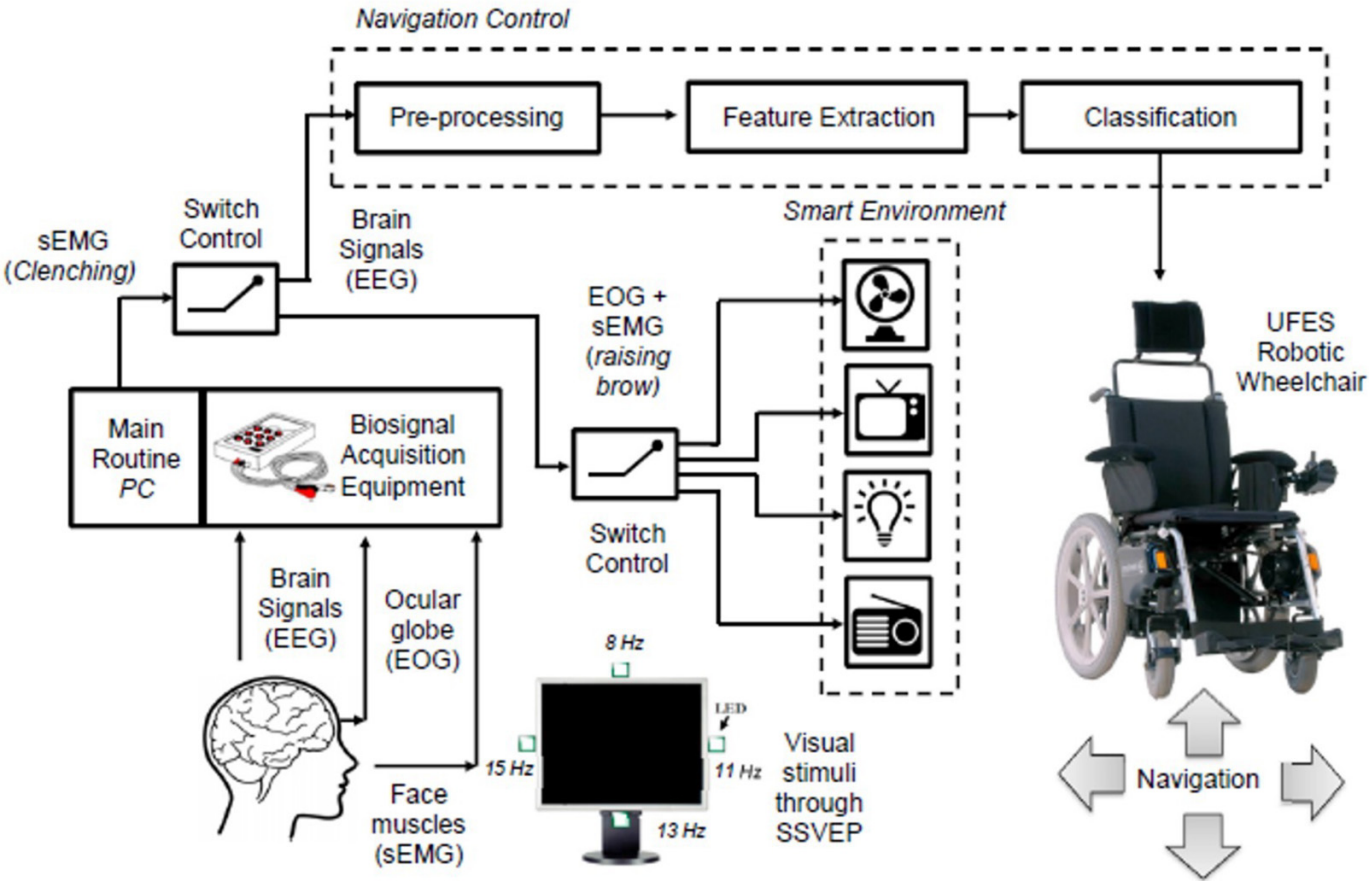
BCI-mediated environmental control. The monitoring and interpretation of EEG, EOG, and EMG data could be used to operate environmental control systems for patients with severe motor impairments. Reproduced with permission from Tello *et al.*, IFAC-PapersOnLine **48**(19), 136–141 (2015). Copyright 2015 IFAC.^153^

Diseases that compromise the function of motor neurons, such as amyotrophic lateral sclerosis, progressive bulbar palsy, and progressive muscular atrophy are characterized by the loss of control over voluntary movement.^149^ In recent years, BCIs have emerged as attractive strategies to restore the ability of these locked-in patients to interact with their surroundings. Multiple EEG-based technologies have been developed to provide patients with severe disabilities with varying degrees of environmental control.^150^ For instance, one of the most widely explored applications of eBCIs is the control of assistive vehicles such as electrical wheelchairs (Fig. 5).^151^ Similarly, EEG-based smart home systems have been developed to provide control over lighting, air-conditioning systems, televisions, and other appliances (Fig. 5).^151–153^

Another advantage provided by technologies that monitor brain activity continuously and noninvasively is the ability to implement these tools in the workplace. The remarkable levels of productivity achieved by modern manufacturing facilities put significant strain on workers, which has led to increases in the incidence of industrial accidents.^154^ Although fatigue, stress, and sleepiness constitute significant factors that impair the performance of workers and increase the risk of accidents, they are particularly hard to measure in real time with conventional methods. Because of this, several groups have explored the development of wEEG-based devices to detect abnormal worker behaviors.^155,156^ This type of eBCIs could be deployed in mass to prevent the occurrence of accidents and enhance occupational safety in factories and other workplaces with high rates of incidents.

Current biometric identifiers are based on physical characteristics, including fingerprints and facile features, as well as voice and iris recognition. More recently, it has been hypothesized that EEG signals acquired during perception or the execution of a mental task could be used for accurate subject identification. EEG-based biometric cryptosystems rely on the comparison of EEG activity from different subjects following the execution of a defined task, such as breathing, finger movement, or singing.^157^ These systems provide remarkably high levels of accuracy and could constitute the basis for future universal biometric security systems that do not rely on a specific body part for subject recognition.

### C. Education, entertainment, and marketing

Technologies that monitor brain activity could be implemented in the classroom to assess different aspects of the learning experience and to study the physiological mechanisms that underlie this process. For instance, previous works have reported that increases in frontal theta activity are indicative of high mental effort and cognitive demand,^158,159^ while decreases in alpha activity are related to long-term memory processing.^160^ These EEG biomarkers could be used to study the effect of cognitive overload and mental fatigue on learning, which could be used to develop more effective subject training methods. In this regard, previous groups have used EEG biomarkers to assess alterations in brain networks induced by cognitive training interventions, which constitutes a promising approach to objectively evaluate the improvement of cognitive functions.^161^ Future eBCIs could be used to assess classroom performance on a per-student basis to design custom learning experiences that are based on the capabilities and limitations of individual subjects.

**FIG. 6.**
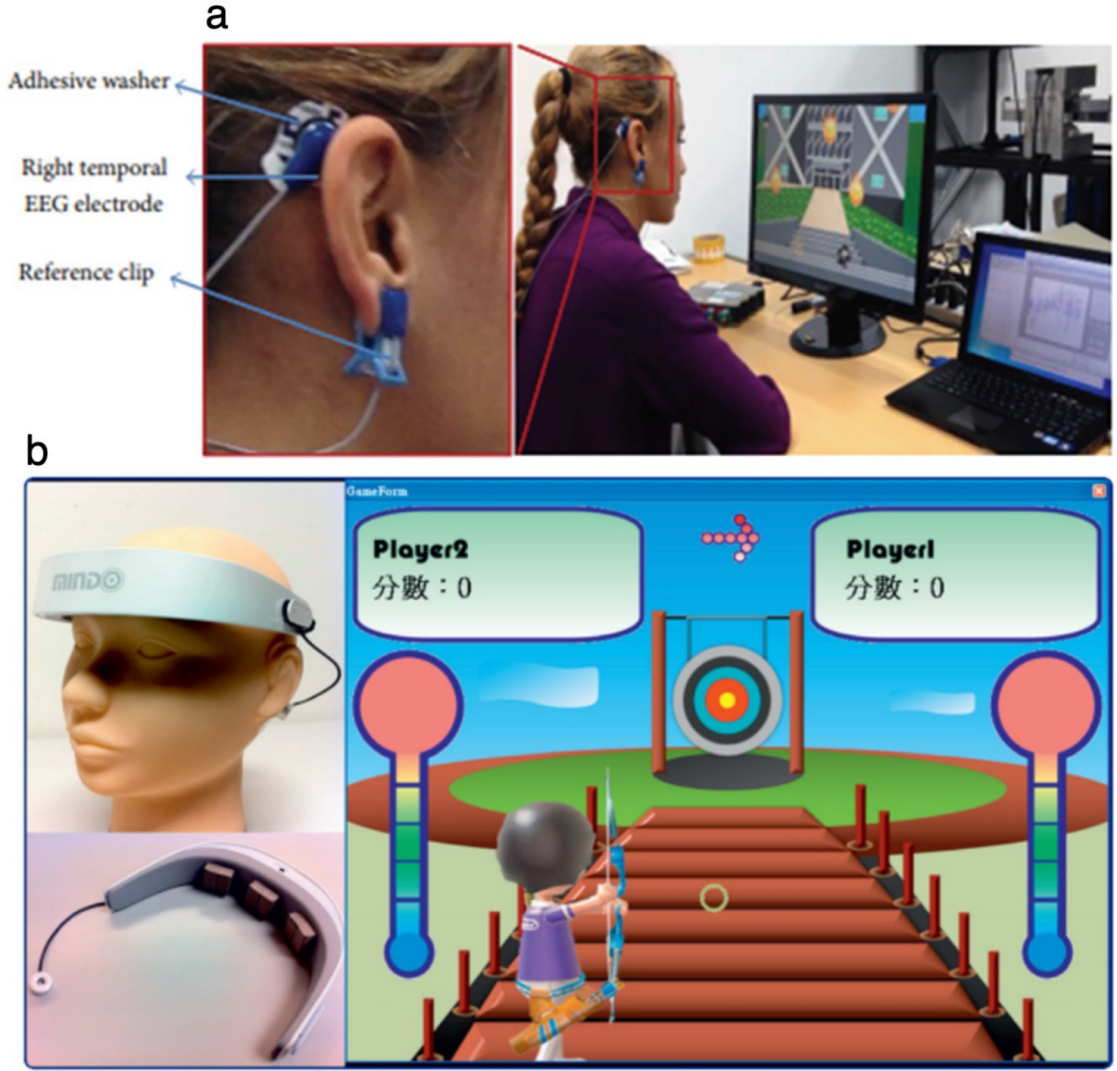
The emerging field of neurogaming. Representative images of (a) neurogaming hardware and (b) user interfaces that rely on eBCI technology. Top panel reproduced with permission from Belkacem *et al.*, Comput. Intell. Neurosci. **2015**, 653639. Copyright 2015 Authors, licensed under a Creative Commons Attribution (CC BY) license.^164^ Bottom panel reproduced with permission from Liao *et al.*, J. Neuroeng. Rehabil. **9**(1), 5 (2012). Copyright 2019 Authors, licensed under a Creative Commons Attribution (CC BY) license.^165^

One of the most active areas of eBCI development outside the clinic is the emerging field of neuro-entertainment. Because of this, a large number of commercially available wEEG devices are targeted toward this market. Following the release of the first consumer-grade EEG system in 2007, NeuroSky (California, USA) developed the game Mindflex in partnership with Mattel (California, USA), which used EEG technology to guide a ball around an obstacle. Since then, a wide variety of consumer-grade wEEG devices have been developed and marketed toward the entertainment and gaming sector.^162,163^ These devices provide basic functionality and are largely based on the use of dry sensors that stream EEG data via Bluetooth to smart devices, such as phones and other wearables. Moreover, additional input sources such as EOG data [Fig. 6(a)]^164^ or user focusing level [Fig. 6(b)]^165^ have been incorporated to provide increased level of control. In addition, eBCIs and gamification-based strategies have also been used for the management of attention deficit hyperactivity disorder (ADHD) through NFB, as well as cognitive skill development for the diagnosis and treatment of cognitive disorders.^166^ Several companies have reported investigating the application of eBCI to the mainstream gaming industry, with suggested applications including monitoring player experience and engagement, syncing gameplay experience with the player's emotional state, reduction of vertigo and motion sickness side effects with virtual reality headsets, and the use of eBCI as an input controller. Gabe Newell, Co-founder and President of Valve, a software/hardware developer and operator of the largest digital distribution platform for PC gaming, is quoted in a recent interview as saying, “If you're in the entertainment business and you're not thinking about this, you're going to be thinking about it a lot more in the future.”^167^

The ability to objectively monitor the attention span of the user has also been used to evaluate audience engagement and predict population level viewership.^168^ Therefore, eBCIs could also be used to tailor viewing experience and to predict the success of programming by identifying cognitive processes that contribute to audience engagement. Similarly, EEG-based technologies have been extensively used in the field of neuromarketing, which uses neuropsychology tools to study consumer behavior.^169–171^ This emerging field studies how different stimuli influence consumer response and the decision-making process involved in the purchasing of products and services. Furthermore, owing to the commercialization of cost-effective wEEG devices, eBCIs hold great promise for the collection of massive amounts of user data to develop more effective marketing strategies.

## VI. FUTURE PERSPECTIVES AND CONCLUSION

With estimated 835 × 10^6^ connected smart devices worldwide, the ongoing surge in consumer wearables has facilitated their use as noninvasive tools for ambulatory health monitoring.^172^ Furthermore, consumer-grade wEEG devices are becoming increasingly accessible and are rapidly catching up to the level of accuracy provided by their clinical counterparts. Recent works have demonstrated that current commercial systems already constitute valuable diagnostic tools, since they can supplement classical neurological examination.^173,174^ In addition, these platforms greatly enhance user experience and comfort, which often constitutes the main limiting factor preventing daily usage by healthy subjects.^175^ Because of this high degree of performance and convenience, wEEG devices are of great importance for the dissemination of eBCI technology among the general population. Moreover, as these systems become progressively more sophisticated, the seamless integration of mind and computers will yield unprecedented ways to interact and engage with the world. However, these breakthroughs could also bring forth several ethical, legal, and social issues that fundamentally alter the interrelationships between humans and technology.

Privacy and agency are two of the most prominent issues often raised by neuroscientific research groups and expert panels convened by independent bodies.^176^ Neural information acquired by eBCIs could be representative of the overall manner in which users think, feel, and behave. In turn, these data could be used to infer different aspects related to user intention, emotional response, and decision making, as well as conscious and unconscious interest. Because of this, eBCIs constitute highly appealing tools to enable large-scale collection of consumer biometric data for commercial research. During the past five years, various tech companies, including Facebook (California, USA), Neuralink (California, USA), Microsoft (Washington, EUA), and multiple dedicated startups have ventured into the development of BCI technologies.^177^ In addition, large government programs have been established to accelerate the emergence and implementation of novel neurotechnologies, such as the U.S. BRAIN initiative^178^ and the Human Brain Project^179^ from the European Commission. Therefore, it is anticipated that the throughput of neural big data will increase significantly in the near future, which raises several concerns regarding sharing and ownership. For instance, once neural information is digitized and stored, it becomes susceptible to unauthorized access or misuse by third parties, and thus, it is critical to ensure that data are handled following strict privacy standards. Furthermore, basic privacy rights are often surrendered by the average user to various service providers, such as search engines, app developers, and social media platforms, without a clear understanding of what is being forfeited. To address this issue, previous authors have suggested that personal neural data should be legally regarded as an organ or tissue, so it is shared exclusively by individuals and unable to be commercialized.^180^ Moreover, sharing of neural data should be carried out through a secure and encrypted process, using informed consent language that clearly specifies who will use the information, for what end, and for how long. Therefore, it is important that researchers, clinicians, manufacturers, patients, and other stakeholders become involved in the development of policies that protect the right to mental privacy, in order to ensure the adequate handling of neural information.

Sense of agency refers to the subjective awareness of control over our own actions and, by extension, over events in the external world.^181^ In recent years, the manner in which personhood can be molded by human–computer interaction has gathered significant public interest. With the increasing use of machine learning and artificial intelligence (AI) for shared-control BCIs, it has been questioned if actuator outputs are invariably and genuinely produced by the user.^14,182^ In addition, owing to the lack of proprioception, the human brain is unable to acknowledge the influence of an external device on itself,^183^ which could potentially compromise autonomy and self-agency. Because of this, users may be liable to mistakenly perceive ownership over behavioral outputs that are generated by the BCI, as well as incorrectly attribute causation to it. For instance, brain stimulation techniques have been shown to trigger changes in demeanor and character traits, which often leads to changes in personal identity.^184^ Subjects undergoing deep brain stimulation (DBS) could develop ambiguities in self-agency and become uncertain of whether their emotional state and behavior are attributable to themselves or to the BCI.^185^ Other studies have shown that impulsive-compulsive behaviors, such as hypersexuality, pathological gambling, compulsive eating, or excessive buying, could be exacerbated following DBS treatment in Parkinson's disease patients.^186^ In turn, this diminished agency and undermined sense of self-could even prevent users from being considered as autonomous agents with decisional capacity. Therefore, the design of mass marketed BCIs should aim to prevent impingements on user autonomy, as well as minimize the risk of dependency and impaired self-perception. In addition, it is crucial to warrant adequate oversight and legislation to protect the right to mental integrity by ensuring the safety of neurotech aimed for the clinic and for the mainstream market.

With the impending widespread adoption of eBCIs and other neurotech platforms for nonclinical applications, additional challenges and risks are bound to emerge.^187^ Owing to the use of eBCIs to improve cognitive function, this technology could potentially be used to produce subjects with artificially enhanced intellectual performance. Based on the right to cognitive liberty, competent adults should be free to benefit from neurotech in any way they see fit, as long as this does not infringe on the liberties of other individuals. However, questions remain regarding the ethics of neuroenhancement of children and cognitively impaired patients by consenting family members. By establishing a direct connection between computers and the human mind, the concepts of memory and learning could also be deeply transformed. Similar to the use of current internet search engines, the capacity for instant recollection of information could raise several questions regarding the fundamental nature of knowledge and education. In addition, the use of neurotech to modulate aspects of personality and emotion could potentially lead to severe health issues triggered by misuse and addiction, analogous to the ongoing opioid epidemic in the U.S.^188^

Despite the inherent risks of neurotech in its gestational state, stringent legislations will ensure that future innovations comply with safety and neuroethical standards. International policies should also guarantee the dissemination of this technology with egalitarianism and accountability to prevent the exacerbation of current socioeconomic inequalities. In addition, further research efforts and public outreach by the scientific community are critical to deter the spread of misinformation, which could delay the advancement of this technology and prevent its full realization. In summary, the potential of BCI technology is beginning to crystalize at a rapid pace, yielding technological solutions that provide tangible benefits to our quality of life. Moving ahead, it is important to pre-emptively establish a clear way forward toward responsible neuroengineering, in hopes of a future where the human mind and technology can come together seamlessly to surpass our own biological limits.

## Data Availability

Data sharing is not applicable to this article as no new data were created or analyzed in this study.
